# Supplementary material for: A Critical View of the Application of the APEX Software (Aqueous Photochemistry of Environmentally-Occurring Xenobiotics) to Predict Photoreaction Kinetics in Surface Freshwaters
Source: Molecules. 2019 Dec 18;25(1):9. doi: 10.3390/molecules25010009 (PMC7017383; doi:10.3390/molecules25010009)
Supplement: Supplementary file 1 [file molecules-25-00009-s001.zip › 3.2.4_gcc-4.4.0.zip!/3.2.4_gcc-4.4.0/3.2.4_gcc-4.4.0/doc/octave/html/interpreter/Representing-Images.html]

Representing Images - Untitled


Next: Plotting on top of Images,
Previous: Displaying Images,
Up: Image Processing


---

### 31.3 Representing Images

In general Octave supports four different kinds of images, gray-scale
images, RGB images, binary images, and indexed images. A gray-scale
image is represented with an M-by-N matrix in which each
element corresponds to the intensity of a pixel. An RGB image is
represented with an M-by-N-by-3 array where each
3-vector corresponds to the red, green, and blue intensities of each
pixel.

The actual meaning of the value of a pixel in a gray-scale or RGB
image depends on the class of the matrix. If the matrix is of class
`double` pixel intensities are between 0 and 1, if it is of class
`uint8` intensities are between 0 and 255, and if it is of class
`uint16` intensities are between 0 and 65535.

A binary image is an M-by-N matrix of class `logical`.
A pixel in a binary image is black if it is `false` and white
if it is `true`.

An indexed image consists of an M-by-N matrix of integers
and a C-by-3 color map. Each integer corresponds to an
index in the color map, and each row in the color map corresponds to
an RGB color. The color map must be of class `double` with values
between 0 and 1.

— Function File: [img, map] = **gray2ind** (I, n)  
> Convert a gray scale intensity image to an Octave indexed image.
> The indexed image will consist of n different intensity values. If not
> given n will default to 64.

— Function File: **ind2gray** (x, map)  
> Convert an Octave indexed image to a gray scale intensity image.
> If map is omitted, the current colormap is used to determine the
> intensities.
>
>
>
> **See also:** gray2ind, rgb2ntsc, image, colormap.

— Function File: [x, map] = **rgb2ind** (rgb)  
— Function File: [x, map] = **rgb2ind** (r, g, b)  
> Convert an RGB image to an Octave indexed image.
>
>
>
> **See also:** ind2rgb, rgb2ntsc.

— Function File: rgb = **ind2rgb** (x, map)  
— Function File: [r, g, b] = **ind2rgb** (x, map)  
> Convert an indexed image to red, green, and blue color components.
> If the colormap doesn't contain enough colors, pad it with the
> last color in the map.
> If map is omitted, the current colormap is used for the conversion.
>
>
>
> **See also:** rgb2ind, image, imshow, ind2gray, gray2ind.

— Function File: **colormap** (map)  
— Function File: **colormap** ("default")  
> Set the current colormap.
>
> `colormap (`map`)` sets the current colormap to map. The
> color map should be an n row by 3 column matrix. The columns
> contain red, green, and blue intensities respectively. All entries
> should be between 0 and 1 inclusive. The new colormap is returned.
>
> `colormap ("default")` restores the default colormap (the
> `jet` map with 64 entries). The default colormap is returned.
>
> With no arguments, `colormap` returns the current color map.
>
>
>
> **See also:** jet.

— Function File: map\_out = **brighten** (map, beta)  
— Function File: map\_out = **brighten** (h, beta)  
— Function File: map\_out = **brighten** (beta)  
> Darkens or brightens the given colormap. If the map argument
> is omitted, the function is applied to the current colormap. The first
> argument can also be a valid graphics handle h, in which case
> `brighten` is applied to the colormap associated with this handle.
>
> Should the resulting colormap map\_out not be assigned, it will be
> written to the current colormap.
>
> The argument beta should be a scalar between -1 and 1,
> where a negative value darkens and a positive value brightens
> the colormap.
>
>
>
> **See also:** colormap.

— Function File: **autumn** (n)  
> Create color colormap. This colormap is red through orange to yellow.
> The argument n should be a scalar. If it
> is omitted, the length of the current colormap or 64 is assumed.
>
>
>
> **See also:** colormap.

— Function File: **bone** (n)  
> Create color colormap. This colormap is a gray colormap with a light
> blue tone. The argument n should be a scalar. If it
> is omitted, the length of the current colormap or 64 is assumed.
>
>
>
> **See also:** colormap.

— Function File: **cool** (n)  
> Create color colormap. The colormap is cyan to magenta. The argument
> n should be a scalar. If it is omitted, the length of the current
> colormap or 64 is assumed.
>
>
>
> **See also:** colormap.

— Function File: **copper** (n)  
> Create color colormap. This colormap is black to a light copper tone.
> The argument n should be a scalar. If it
> is omitted, the length of the current colormap or 64 is assumed.
>
>
>
> **See also:** colormap.

— Function File: **flag** (n)  
> Create color colormap. This colormap cycles through red, white, blue
> and black. The argument n should be a scalar. If it
> is omitted, the length of the current colormap or 64 is assumed.
>
>
>
> **See also:** colormap.

— Function File: **gray** (n)  
> Return a gray colormap with n entries corresponding to values from
> 0 to n-1. The argument n should be a scalar. If it is
> omitted, the length of the current colormap or 64 is assumed.

— Function File: **hot** (n)  
> Create color colormap. This colormap is black through dark red, red,
> orange, yellow to white. The argument n should be a scalar. If it
> is omitted, the length of the current colormap or 64 is assumed.
>
>
>
> **See also:** colormap.

— Function File: **hsv** (n)  
> Create color colormap. This colormap is red through yellow, green,
> cyan, blue, magenta to red. It is obtained by linearly varying the
> hue through all possible values while keeping constant maximum
> saturation and value and is equivalent to
> `hsv2rgb ([linspace(0,1,N)', ones(N,2)])`.
>
> The argument n should be a scalar. If it is omitted, the
> length of the current colormap or 64 is assumed.
>
>
>
> **See also:** colormap.

— Function File: **jet** (n)  
> Create color colormap. This colormap is dark blue through blue, cyan,
> green, yellow, red to dark red. The argument n should be a scalar.
> If it is omitted, the length of the current colormap or 64 is assumed.
>
>
>
> **See also:** colormap.

— Function File: **ocean** (n)  
> Create color colormap. The argument n should be a scalar. If it
> is omitted, the length of the current colormap or 64 is assumed.

— Function File: **pink** (n)  
> Create color colormap. This colormap gives a sepia tone on black and
> white images. The argument n should be a scalar. If it
> is omitted, the length of the current colormap or 64 is assumed.
>
>
>
> **See also:** colormap.

— Function File: **prism** (n)  
> Create color colormap. This colormap cycles trough red, orange, yellow,
> green, blue and violet. The argument n should be a scalar. If it
> is omitted, the length of the current colormap or 64 is assumed.
>
>
>
> **See also:** colormap.

— Function File: **rainbow** (n)  
> Create color colormap. This colormap is red through orange, yellow, green,
> blue to violet. The argument n should be a scalar. If it
> is omitted, the length of the current colormap or 64 is assumed.
>
>
>
> **See also:** colormap.

— Function File: **spring** (n)  
> Create color colormap. This colormap is magenta to yellow.
> The argument n should be a scalar. If it
> is omitted, the length of the current colormap or 64 is assumed.
>
>
>
> **See also:** colormap.

— Function File: **summer** (n)  
> Create color colormap. This colormap is green to yellow.
> The argument n should be a scalar. If it
> is omitted, the length of the current colormap or 64 is assumed.
>
>
>
> **See also:** colormap.

— Function File: **white** (n)  
> Create color colormap. This colormap is completely white.
> The argument n should be a scalar. If it
> is omitted, the length of the current colormap or 64 is assumed.
>
>
>
> **See also:** colormap.

— Function File: **winter** (n)  
> Create color colormap. This colormap is blue to green.
> The argument n should be a scalar. If it
> is omitted, the length of the current colormap or 64 is assumed.
>
>
>
> **See also:** colormap.

— Function File: **contrast** (x, n)  
> Return a gray colormap that maximizes the contrast in an image. The
> returned colormap will have n rows. If n is not defined
> then the size of the current colormap is used instead.
>
>
>
> **See also:** colormap.

An additional colormap is `gmap40`. This code map contains only
colors with integer values of the red, green and blue components. This
is a workaround for a limitation of gnuplot 4.0, that does not allow the color of
line or patch objects to be set, and so `gmap40` is useful for
gnuplot 4.0 users, and in particular in conjunction with the bar,
barh or contour functions.

— Function File: **gmap40** (n)  
> Create a color colormap. The colormap is red, green, blue, yellow,
> magenta and cyan. These are the colors that are allowed with patch
> objects using gnuplot 4.0, and so this colormap function is specially
> designed for users of gnuplot 4.0. The argument n should be
> a scalar. If it is omitted, a length of 6 is assumed. Larger values
> of n result in a repetition of the above colors
>
>
>
> **See also:** colormap.

You may use the `spinmap` function to cycle through the colors in
the current colormap, displaying the changes for the current figure.

— Function File: **spinmap** (t, inc)  
> Cycle the colormap for t seconds with an increment
> of inc. Both parameters are optional. The default cycle time
> is 5 seconds and the default increment is 2.
>
> A higher value of inc causes a faster cycle through the
> colormap.
>
>
>
> **See also:** gca, colorbar.
